# Associations between novel anthropometric measures and the prevalence of hypertension among 45,853 adults: A cross-sectional study

**DOI:** 10.3389/fcvm.2022.1050654

**Published:** 2022-11-03

**Authors:** Li-Da Wu, Chao-Hua Kong, Yi Shi, Jun-Xia Zhang, Shao-Liang Chen

**Affiliations:** Department of Cardiology, Nanjing First Hospital, Nanjing Medical University, Nanjing, China

**Keywords:** hypertension, anthropometric measurements, abdominal obesity, NHANES, restricted cubic spline

## Abstract

**Aims:**

Traditional anthropometric measures, including body mass index (BMI), are insufficient for evaluating the risk of hypertension. We aimed to investigate the association between novel anthropometric indices and hypertension risk in a large population in the United States.

**Methods:**

Forty-five thousand eight hundred fifty-three participants from the National Health and Nutrition Examination Survey (NHANES) (1999–2018) were enrolled. Social demographic information, lifestyle factors, blood biochemical measurements and anthropometric indices, including body weight, body mass index (BMI), waist circumference, waist-to-height ratio (WtHR), conicity index (CI), a body shape index (ABSI), body roundness index (BRI) and lipid accumulation product (LAP) were collected. Multivariable logistic regression and restricted cubic spline were adopted to investigate the associations between hypertension risk and anthropometric indices. We also performed receiver operating characteristic (ROC) curve analyses to further evaluate the discriminatory powers of anthropometric measurements for screening hypertension risk. Moreover, participants were randomly assigned to the training group and the validation group in a ratio of 3 to 1. A nomogram model based on anthropometric measures was established and validated in the training group and validation group, respectively.

**Results:**

All of the anthropometric measurements investigated were positively and independently associated with the hypertension risk. Among all anthropometric indices, per-SD increment in ABSI had the highest OR (OR: 3.4; 95% CI: 2.73–4.24) after adjusting for age, sex, race/ethnicity, education, smoking, drinking, diabetes, and eGFR. Moreover, results from restricted cubic splines revealed the non-linear association between anthropometric measurements and hypertension risk. In ROC analyses, CI had superior discriminatory power for hypertension (area under the curve: 0.71; 95% CI: 0.706–0.715; optimal cutoff value: 1.3) compared with other indices. Nomogram model based on age, sex, diabetes, CI and LAP showed favorable predicting ability of hypertension risk with an AUC (95% CI) in training group of 80.2% (79.7–80.6%), and the AUC (95% CI) in validation group was 79.5% (78.3–80.1%). Meanwhile, calibration plot showed good consistency.

**Conclusions:**

Anthropometric measurements including BMI, WtHR, CI, ABSI, BRI and LAP are closely associated with hypertension risk in the present study. For better prevention and treatment of hypertension, more attention should be paid to anthropometric indices, especially novel anthropometric indices.

## Introduction

Hypertension and its complications pose an important health care burden worldwide. Globally, hypertension affects about one billion people and is the leading cause of cardiovascular death and morbidity (1). Obesity is one of the major risk factors for hypertension, which in turn causes cardiovascular diseases and other complications. The mechanisms by which obesity leads to hypertension is thought to be related to inflammation caused by excessive adiposity accumulation, especially visceral fat deposition (2).

Anthropometric indices, including body weight, body mass index (BMI), waist circumference and *etc*, are the main tools used to diagnose and screen obese individuals. They are widely used in clinical practice and have the advantages of non-invasive, effective and easy to implement (3). BMI is the mostly used anthropometric measurement for general obesity at present. However, it does not accurately reflect actual obesity status and the distribution of body fat. Recent studies have already proven that abdominal obesity is more harmful than general obesity. It is necessary to combine BMI and waist circumference in the assessment of obesity, which is helpful for the evaluation of different obesity patterns (4). Although using waist circumference to assess abdominal obesity and abdominal adiposity accumulation is objective, it still ignores the effect of height. Waist-to-height ratio (WtHR) is another effective index to evaluate abdominal obesity. In consideration of individual height, WtHR has the advantages of small variation, relatively stable among different populations. It has better application value especially for children and adolescents. Nevertheless, recent evidence has also pointed out that WtHR is not always the optimal measurement (5).

There are several novel anthropometric measurements proposed to better evaluate obesity in the past few decades. Conicity index (CI), a composite anthropometric index, calculated based on body weight, body height (BH), and waist circumference, was firstly proposed in 1991 to better determine the degree of central obesity and accumulation of abdominal adiposity (6). CI has already been widely used to assess the risk of developing different diseases, recent studies demonstrated that CI could better predict the changes in lipid profiles and cardiovascular risk among adolescents than waist circumference and WtHR (7, 8). A Body Shape Index (ABSI) and body roundness index (BRI) are also novel anthropometric measurements created to estimate both degree of abdominal obesity and the amount visceral adipose. Results from a recent meta-analysis showed that ABSI and BRI both had good discriminatory ability for diabetes in adults from multiracial populations (9). But the associations between ABSI, BRI and hypertension need to be further explored. Lipid accumulation product (LAP), based on a combination of waist circumference and serum triglycerides (TG), is another useful tool to reflect the levels of fat deposition. A cross-sectional study enrolled 2,500 participants from China showed that higher LAP was associated with insulin resistance and increased risk of diabetes (10). In addition, LAP is positively correlated with oxidative stress and inflammation, which plays an important role in the occurrence and development of hypertension (11).

We conducted this cross-sectional study to explore the association between eight often used anthropometric measurements and the prevalence of hypertension in a large multiracial cohort in the US. We also determined and compared the discriminatory ability of anthropometric indices as instruments for screening hypertension risk. Moreover, the optimal cutoff values of these measurements were calculated to help health care professionals assess hypertension risk.

## Methods

### Study design and participants

National Health and Nutrition Examination Survey (NHANES) is a program designed to measure the health and nutrition status of adults and children in the United States (12). The method of “stratified multistage probability sampling” was adopted to screen out representative participants in NHANES survey. Detailed methods are described in the NHANES website (http://www.cdc.gov/nchs/nhanes.htm). All participants enrolled in NHANES provided written informed consent, and the whole procedures were approved by the Institutional Review Board of the Centers for Disease Control and Prevention. An analysis of 10 consecutive NHANES circles from 1999/2000 to 2017/2018 was conducted in the present study. The exclusion criteria were as follows: (1) age <18 or ≥80 years, (2) pregnancy and missing anthropometric data, (3) estimated glomerular filtration rate (eGFR) <60 ml/min/1.73 m^2^.

### Anthropometric measurements

Experienced examiners measured basic anthropometric measurements, including body weight, BH and waist circumference, at the mobile examination center with standardized techniques and equipment. Waist circumference, an index for abdominal obesity, was measured at the superior border of the iliac crests. BMI, WtHR, CI, ABSI, BRI and LAP were calculated according to previous published formulae (13) as followed:


(1) $$\begin{array}{lll} BMI & = & BW\;\left(kg\right)/BH^{2}\left(m\right) \end{array}$$

(2) $$\begin{array}{lll} WtHR & = & WC\left(cm\right)/BH\left(m\right) \end{array}$$

(3) $$\begin{array}{lll} CI & = & \frac{WC\left(m\right)}{0.109\;\times \sqrt{\frac{BW\;\left(kg\right)}{BH\left(m\right)}}} \end{array}$$

(4) $$\begin{array}{lll} ABSI & = & \;WC\left(cm\right)/BMI^{\frac{2}{3}}\left(kg/m^{2}\right)\times BH^{\frac{1}{2}}\left(m\right) \end{array}$$

(5) $$\begin{array}{lll} BRI & = & 364.2-365.5\times \sqrt{1-{\left(\frac{\frac{WC\left(m\right)}{2\pi }}{0.5\times BH\left(m\right)}\right)}^{2}} \end{array}$$

(6) $$\begin{array}{l} \begin{array}{lll} LAP & = & \left(WC(cm)-65\right) \end{array}\\ \;\begin{array}{ll} \times & TG(mmol/L)\;in\;male\;individuals \end{array} \end{array}$$

(7) $$\begin{array}{l} \begin{array}{lll} LAP & = & \;(WC(cm)-65) \end{array}\\ \;\begin{array}{ll} \times & TG(mmol/L)\;in\;female\;individuals \end{array} \end{array}$$

### Definition of hypertension

The blood pressure was recorded by a trained examiner according to the protocol of blood pressure measurement released by the American Heart Association. The average systolic blood pressure (SBP) and diastolic blood pressure (DBP) of three consecutive measurements was obtained and reported. It is the same as the previous published researches on the analysis of NHANES database (14), participants meeting one or more of the following criteria were considered to have hypertension: (1) average SBP ≥ 140 mmHg or average DBP ≥ 90 mmHg; (2) individuals with prescribed antihypertensive medications; (3) self-reported hypertension. The criteria of 140/90 mmHg refers to the guideline of International Society of Hypertension (15).

### Covariates

Age, sex, race/ethnicity, and education levels were obtained from the demographic questionnaires. Diabetes history, alcohol consumption and smoking status were adopted from the health questionnaires. Age (years) was used as a continuous variable. Sex was classified as male or female. Race/ethnicity was classified as Mexican American, non-Hispanic White, non-Hispanic Black, other Hispanic, and others. Smoking and drinking status were categorized as yes or no. Education was divided into three levels: below high school, high school and above high school. After at least 8 h of an overnight fast, blood samples were collected and used to examine the levels of TG, total cholesterol (TC), low-density lipoprotein cholesterol (LDL-C), high-density lipoprotein cholesterol (HDL-C), red blood cells (RBC), white blood cells (WBC), platelet (PLT), neutrophil (NE), lymphocyte (LY), hemoglobin, glycosylated hemoglobin (HbA1c), fasting blood glucose (FBG). The estimated glomerular filtration rate (eGFR) was calculated according to the Chronic Kidney Disease Epidemiology Collaboration creatinine equation. NHANES website provided the detailed procedures in collecting blood biochemical measurements.

### Statistical methods

For descriptive statistics, continuous variables are presented as the mean ± standard deviation (SD), and categorical variables are described as counts with percentages. We compared baseline characteristics among individuals with and without hypertension based on independent *t*-tests, chi-square test, and Mann–Whitney *U* test. Multivariable logistic regression was adopted to investigate the association between hypertension risk and anthropometric indices after adjusting for confounding factors (age, sex, race/ethnicity, education, smoking, drinking, diabetes, and eGFR). Odds ratios (ORs) were logarithm-transformed to represent the rate of increase. All anthropometric variables were z-standardized and treated as continuous variables. Restricted cubic spline analysis (with 3 knots) was used to evaluate the non-linear associations between anthropometric measurements and hypertension risk, the median value of each anthropometric measurement was used as a reference. Subgroup analysis, stratified by age, sex, BMI and race/ethnicity, were conducted to evaluate the heterogeneity. Receiver operating characteristic (ROC) curves was used to evaluate the discriminative powers of anthropometric measurements in identifying individuals with hypertension. According to the World Health Organization (WHO) guidelines, obesity was defined as BMI ≥ 30.0 kg/m^2^; abdominal obesity was defined as waist circumference ≥102 cm for men and waist circumference ≥80 cm for women. We further examined whether integrating BMI with other anthropometric measures (mean value) could better assess the risk of hypertension. Correlation analysis between the two anthropometric measurements was conducted based on Spearman method. Using “car” package in R software, all of the participants included in the present study were randomly assigned to the training group and the validation group in a ratio of 3 to 1. To further evaluate the discriminating ability of novel anthropometric indices in screening hypertension, nomogram model was created. The accuracy of nomogram model in training group and validation group was also evaluated through ROC curves. Calibration plot of by bootstrap validation with 1,000 re-samplings was adopted used to evaluate the consistency in the training group. Considering the application of medication could have an impact on the blood pressure of participants, individuals who received any drug treatment were excluded in sensitive analysis. The sample size in sensitivity analyses was 22,817. Multiple imputation was used to fill missing covariates to avoid the selection bias due to excluding participants with missing data. A two-sided *P*-value <0.05 was considered significant. All statistical analyses were conducted using R software (16) (version 4.1.6).

## Results

### Characteristics of the study population

**Figure 1:**
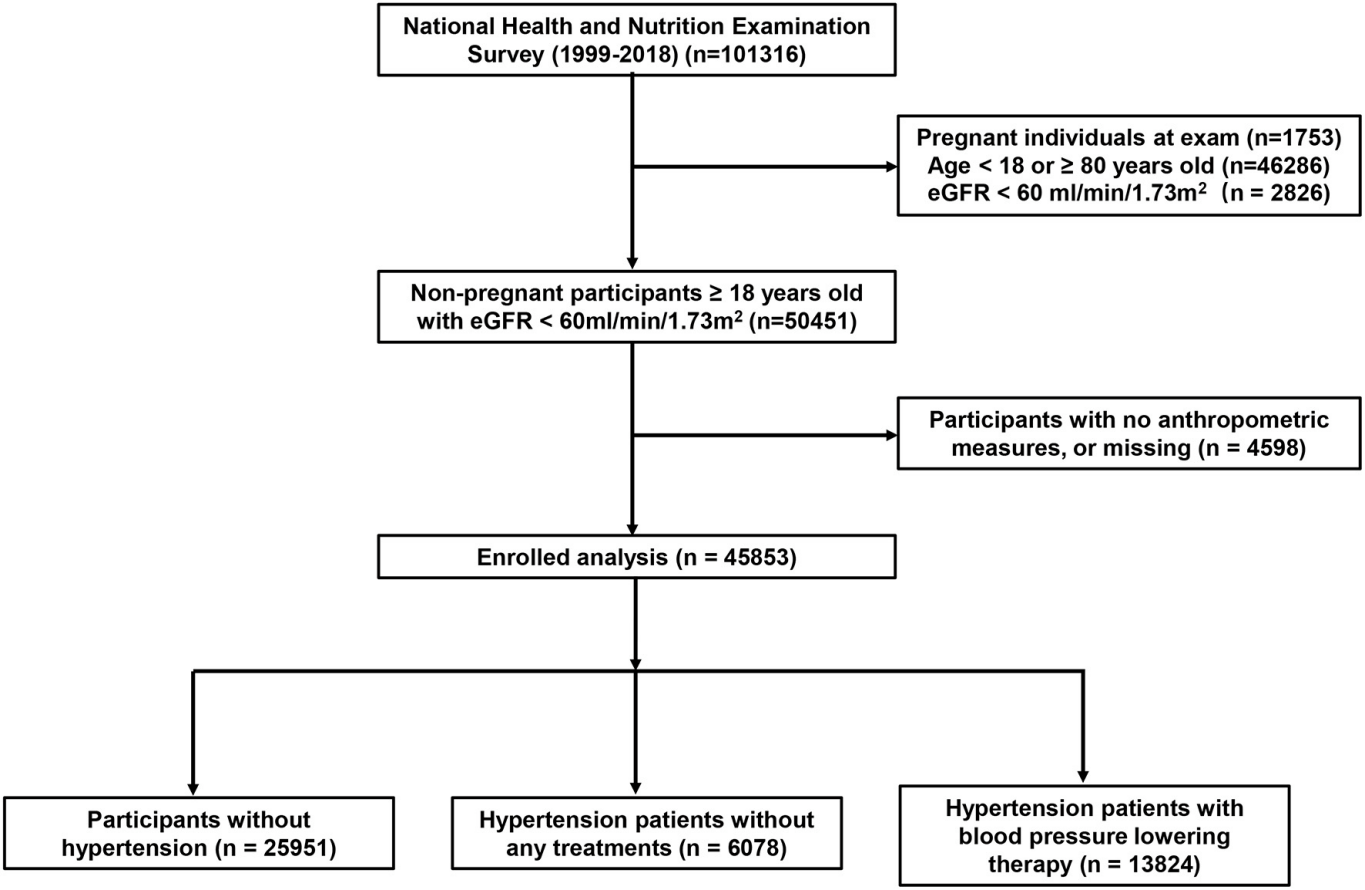
Flowchart of the study population. eGFR, estimated glomerular filtration rate.

Forty-five thousand eight hundred fifty-three participants from NHANES (1999–2018) was enrolled in the present study, flowchart of the study population was shown in Figure 1. Differences was observed in the demographic and clinical data at baseline among participants with hypertension and participants without hypertension (Table 1). Compared with participants without hypertension, the average age (37.01 ± 14.9 vs. 53.6 ± 15.05) and the proportion of male (47.7 vs. 53.1%) were higher in participants with hypertension. Participants with hypertension also had higher proportion of smokers (39.8 vs. 49.6%) and lower educational levels. There was no difference in the proportion of alcohol users among participants with hypertension and without hypertension. Moreover, eGFR of participants with hypertension were lower comparing with participants without hypertension (106.50 ± 18.87 vs. 93.08 ± 17.99). Eight often used anthropometric parameters involved in the present study, including body weight, BMI, waist circumference, WtHR, CI, ABSI, BRI and LAP, were all elevated in participants with hypertension. Supplementary Table S1 shows the detailed baseline characteristics of enrolled participants grouped by sex. Overall, male individuals presented higher body weight (86.13 ± 20.28 vs. 75.50 ± 20.43, *P* < 0.001), waist circumference (99.06 ± 16.02 vs. 95.61 ± 16.79, *P* < 0.001), WtHR (49.23 ± 10.79 vs. 46.85 ± 12.16, *P* < 0.001), CI (1.30 ± 0.09 vs. 1.29 ± 0.09, *P* < 0.001), ABSI (0.09 ± 0.003 vs. 0.08 ± 0.003, *P* < 0.001), and lower BMI (28.2 ± 5.96 vs. 29.13 ± 7.45, *P* < 0.001), BRI (4.89 ± 2.05 vs. 5.54 ± 2.51, *P* < 0.001). There was no difference between male individuals and female individuals in LAP (55.06 ± 4.84 vs 55.06 ± 4.4, *P* < 0.001).

**Table 1:** Baseline characteristics.

**Variables**	**Overall** **(*n* = 45,853)**	**Non-hypertension** **(*n* = 25,951)**	**Hypertension** **(*n* = 19,902)**	***P*-value**
Age, years	44.21 ± 17.08	37.01 ± 14.90	53.6 ± 15.05	<0.001***
Sex-male, *n* (%)	22,962 (50.1)	12,387 (47.7)	10,575 (53.1)	<0.001***
Race, *n* (%)				<0.001***
Non-Hispanic White	18,372 (40.1)	10,351 (39.9)	8,021 (40.3)	
Non-Hispanic Black	10,120 (22.1)	4,812 (18.5)	5,308 (26.7)	
Mexican American	8,953 (19.5)	5,604 (21.6)	3,349 (16.8)	
Other Hispanic	3,958 (8.6)	2,385 (9.2)	1,573 (7.9)	
Other	4,450 (9.7)	2,799 (10.8)	1,651 (8.3)	
Smoking, *n* (%)	20,207 (44.1)	10,334 (39.8)	9,873 (49.6)	<0.001***
Drinking, *n* (%)	27,948 (61.0)	15,880 (61.2)	12,068 (60.6)	0.231
Education level, *n* (%)				<0.001***
Below high school	12,447 (27.1)	6,628 (25.5)	5,819 (29.2)	
High school	10,952 (23.9)	6,070 (23.4)	4,882 (24.5)	
Above high school	22,454 (49.0)	13,253 (51.1)	9,201 (46.2)	
SBP, mmHg	121.96 ± 17.58	112.31 ± 9.27	134.54 ± 17.90	<0.001***
DBP, mmHg	70.91 ± 11.69	67.39 ± 9.16	75.51 ± 12.97	<0.001***
Diabetes, *n* (%)	6,390 (13.9)	1,600 (6.2)	4,790 (24.1)	<0.001***
FBG, mmol/L	5.87 ± 1.90	5.56 ± 1.47	6.27 ± 2.28	<0.001***
HbA1c, %	5.64 ± 1.05	5.43 ± 0.82	5.92 ± 1.23	<0.001***
eGFR, ml/min/1.73 m^2^	100.68 ± 19.65	106.50 ± 18.87	93.08 ± 17.99	<0.001***
Anthropometric measures
Body weight, kg	80.82 ± 21.04	76.56 ± 18.97	86.38 ± 22.26	<0.001***
BMI, kg/m^2^	28.66 ± 6.76	27.16 ± 6.12	30.62 ± 7.05	<0.001***
Waist circumference, cm	97.33 ± 16.50	92.61 ± 15.13	103.50 ± 16.18	<0.001***
WtHR	48.04 ± 11.55	45.52 ± 10.42	51.33 ± 12.12	<0.001***
CI	1.29 ± 0.09	1.26 ± 0.09	1.33 ± 0.09	<0.001***
ABSI	0.08 ± 0.003	0.08 ± 0.004	0.08 ± 0.003	<0.001***
BRI	5.22 ± 2.31	4.58 ± 2.05	6.04 ± 2.38	<0.001***
LAP	55.06 ± 4.63	47.70 ± 4.17	64.65 ± 5.01	<0.001***
TG, mmol/L	1.48 ± 1.20	1.42 ± 1.13	1.55 ± 1.28	<0.001***
TC, mmol/L	5.01 ± 1.08	4.88 ± 1.04	5.17 ± 1.11	<0.001***
LDL-C, mmol/L	2.98 ± 1.00	2.86 ± 0.97	3.12 ± 1.02	<0.001***
HDL-C, mmol/L	1.36 ± 0.40	1.37 ± 0.39	1.34 ± 0.42	<0.001***
RBC, × 109/L	4.74 ± 0.49	4.74 ± 0.48	4.75 ± 0.50	0.05
WBC, × 109/L	7.19 ± 2.21	7.12 ± 2.10	7.29 ± 2.34	<0.001***
NE, × 109/L	4.21 ± 1.67	4.16 ± 1.65	4.27 ± 1.69	<0.001***
Monocyte, × 109/L	0.55 ± 0.19	0.54 ± 0.18	0.56 ± 0.20	<0.001***
LY, × 109/L	2.19 ± 0.98	2.18 ± 0.80	2.20 ± 1.18	0.007**
PLT, × 106/L	255.58 ± 66.18	256.58 ± 64.53	254.27 ± 68.26	<0.001***
Hemoglobin, g/L	14.26 ± 1.51	14.24 ± 1.50	14.29 ± 1.52	0.002**

Variables are presented as the mean ± SD (continuous) or number with percent (categorical). SD, standard deviation; SBP, systolic blood pressure; DBP, diastolic blood pressure; FBG, fasting blood glucose; HbA1c, glycated hemoglobin; eGFR, estimated glomerular filtration rate; BMI, body mass index; WtHR, waist-to-height ratio; CI, conicity index; ABSI, a body shape index; BRI, body round index; LAP, lipid accumulation product; TG, triglycerides; TC, total cholesterol; LDL-C, low-density lipoprotein cholesterol; HDL-C, high-density lipoprotein cholesterol; RBC, red blood cells; WBC, white blood cells; NE, neutrophils; LY, lymphocytes; PLT, platelets. ***P-value < 0.001, **P-value < 0.01.

### Associations between eight anthropometric measures and hypertension

**Figure 2:**
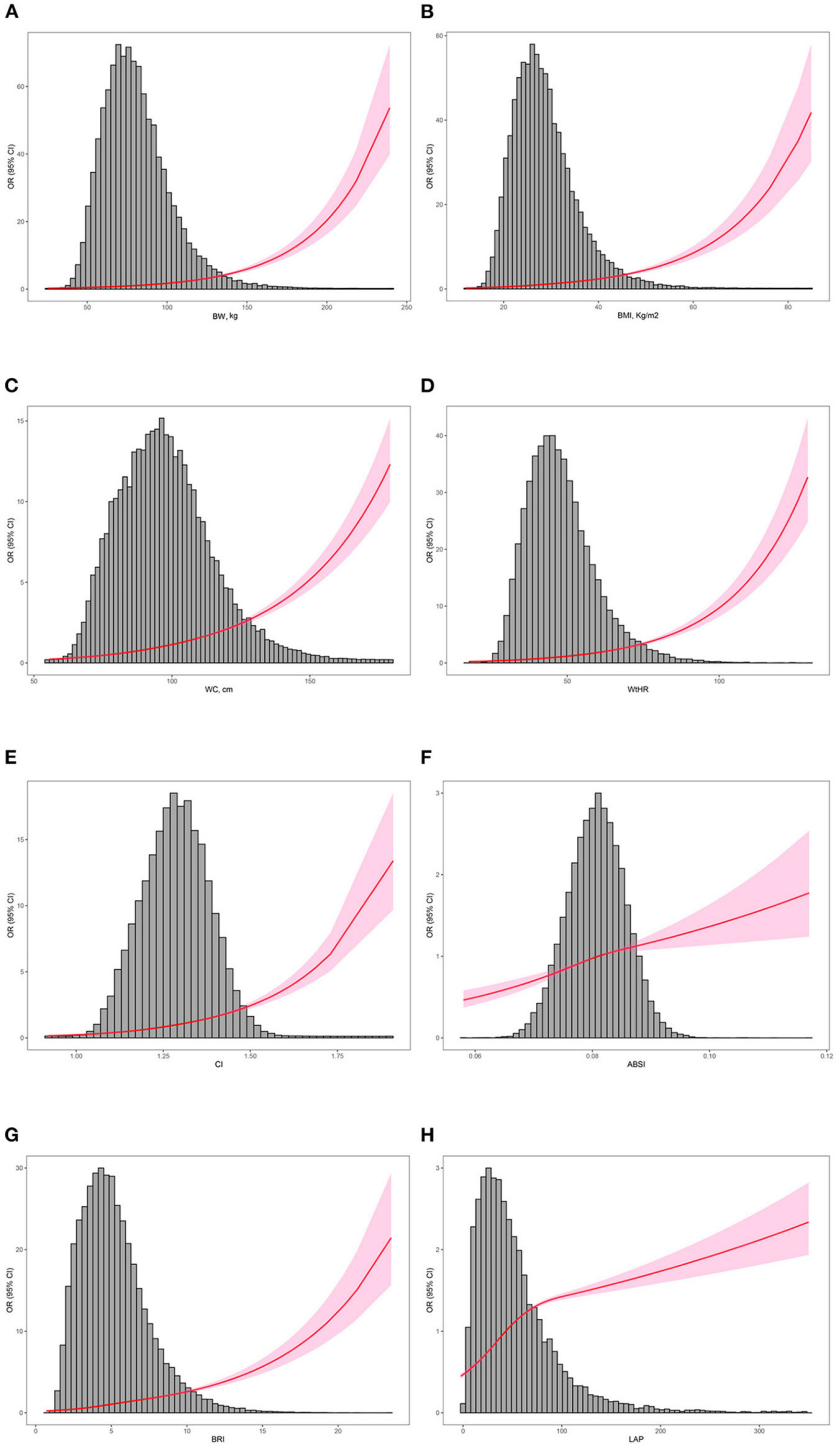
Distribution of the anthropometric measurements of participants enrolled and restricted cubic splines. The distribution histogram is represented in the background and restricted cubic spline analyses were adjusted for age, sex, race/ethnicity, education, smoking, drinking, diabetes, and eGFR. **(A)** Association between BW and the risk of hypertension; **(B)** association between BMI and the risk of hypertension; **(C)** association between WC and the risk of hypertension; **(D)** association between WtHR and the risk of hypertension; **(E)** association between CI and the risk of hypertension; **(F)** association between ABSI and the risk of hypertension; **(G)** association between BRI and the risk of hypertension; **(H)** association between LAP and the risk of hypertension. BW, body weight; BMI, body mass index; WC, waist circumference; WtHR, waist-to-height ratio; CI, conicity index; ABSI, a body shape index; BRI, body round index; LAP, lipid accumulation product.

A positive correlation was found between all of the anthropometric measures studied and the prevalence of hypertension (Table 2). In the non-adjusted model I, ABSI had the highest OR (per-SD increment) (OR: 3.6; 95% CI: 3.03–4.29; *P* < 0.001) among all anthropometric measures. After adjusting for cofounders of age, sex, race/ethnicity, education, smoking, drinking, diabetes, and eGFR, body weight (OR: 1.68; 95% CI: 1.65–1.72; *P* < 0.001), BMI (OR: 1.68; 95% CI: 1.65–1.71; *P* < 0.001), waist circumference (OR: 1.71; 95% CI: 1.68–1.75; *P* < 0.001), WtHR (OR: 1.68; 95% CI: 1.65–1.72; *P* < 0.001), CI (OR: 1.51; 95% CI: 1.48–1.54; *P* < 0.001), ABSI (OR: 3.4; 95% CI: 2.73–4.24; *P* < 0.001), BRI (OR: 1.7; 95% CI: 1.66–1.74; *P* < 0.001) and LAP (OR: 1.31; 95% CI: 1.29–1.34; *P* < 0.001) were still associated with hypertension in the fully adjusted model II. In addition, we adopted restricted cubic splines to further investigate the associations between hypertension and anthropometric measures. Results of restricted cubic splines also demonstrated that body weight, BMI, waist circumference, WtHR, CI, ABSI, BRI and LAP were all positively corelated with the occurrence of hypertension, and in a non-linear pattern. The risk of hypertension increased rapidly with the increase of these parameters, especial in the upper quantile (Figure 2). We also carried out subgroup analyses, stratified by age (<60 and ≥60 years), sex (male and female), race/ethnicity (White, Black, and others) and BMI (<30 and ≥30 kg/m^2^), after adjusting for age, sex, race/ethnicity, education, smoking, drinking, diabetes, and eGFR (Supplementary Table S2). All of the eight anthropometric measures were more closely associated with hypertension in younger participants and those with a BMI <30 kg/m^2^ (*P* interaction <0.001). The increment in BMI, waist circumference, WtHR, CI, ABSI and BRI were more strongly correlated with hypertension in male individuals comparing with female individuals (*P* interaction < 0.05). However, LAP was more saliently associated with risk of hypertension in female individuals. We also found that the non-Hispanic White population was more sensitive to these eight anthropometric measures than the non-Hispanic Black and other populations (*P* interaction < 0.05).

**Table 2:** Logistic regression analysis of anthropometric measures and hypertension.

	**Non-adjusted model**		**Model I**		**Model II**	
	**OR [95% CI]**	***P*-value**	**OR [95% CI]**	***P*-value**	**OR [95% CI]**	***P*-value**
Body weight	1.64 [1.61, 1.67]	<0.001***	1.74 [1.70, 1.78]	<0.001***	1.68 [1.65, 1.72]	<0.001***
BMI	1.73 [1.7, 1.76]	<0.001***	1.75 [1.71, 1.78]	<0.001***	1.68 [1.65, 1.71]	<0.001***
Waist circumference	2.08 [2.04, 2.12]	<0.001***	1.79 [1.75, 1.82]	<0.001***	1.71 [1.68, 1.75]	<0.001***
WtHR	1.71 [1.68, 1.74]	<0.001***	1.74 [1.71, 1.78]	<0.001***	1.68 [1.65, 1.72]	<0.001***
CI	2.17 [2.13, 2.21]	<0.001***	1.58 [1.55, 1.62]	<0.001***	1.51 [1.48, 1.54]	<0.001***
ABSI	3.6 [3.03, 4.29]	<0.001***	3.02 [2.53, 3.94]	<0.001***	3.4 [2.73, 4.24]	<0.001***
BRI	2.03 [1.99, 2.07]	<0.001***	1.78 [1.74, 1.81]	<0.001***	1.7 [1.66, 1.74]	<0.001***
LAP	1.48 [1.45, 1.51]	<0.001***	1.37 [1.34, 1.39]	<0.001***	1.31 [1.29, 1.34]	<0.001***

Data are presented as OR [95% CI], and P-values for per SD increment. Model I adjusted for age, sex, and race/ethnicity. Model II adjusted for age, sex, race/ethnicity, smoking, drinking, education levels, diabetes and eGFR. OR, odds ratio; CI, confidence interval; SD, standard deviation; BMI, body mass index; WtHR, waist-to-height ratio; CI, conicity index; ABSI, a body shape index; BRI, body round index; LAP, lipid accumulation product. ***P-value <0.001.

### Discrimination ability of different anthropometric measures

**Figure 3:**
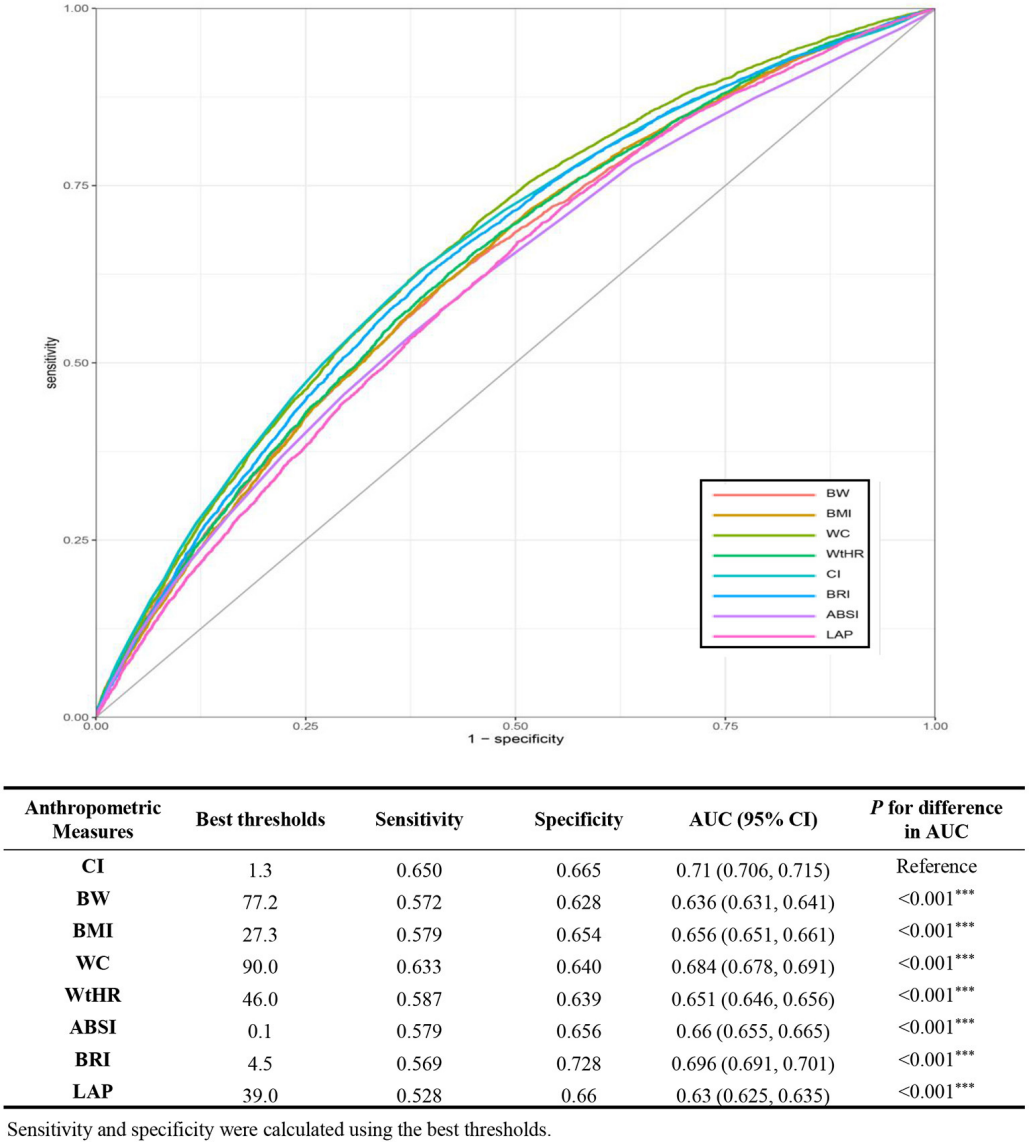
ROC curves of anthropometric indices for discriminating hypertension. ROC, receiver operating characteristic; AUC, area under the curve; BW, body weight; BMI, body mass index; WC, waist circumference; WtHR, waist-to-height ratio; CI, conicity index; ABSI, a body shape index; BRI, body round index; LAP, lipid accumulation product.

ROC curves and area under the curve (AUC) were used to evaluate the abilities of different anthropometric measures in discriminating individuals with hypertension (Figure 3). Results show that CI had the best diagnostic abilitycompared with other seven anthropometric measures (*P* < 0.001) with an AUC of 0.71 (95% CI: 0.706–0.715), in the optimal cutoff value of 1.3, the sensitivity was 0.65 and the specificity was 0.665.

Moreover, body weight, BMI, waist circumference, WtHR, ABSI, BRI and LAP all showed favor AUC values. Moreover, subgroup ROC curve analyses stratified by BMI (<30 and ≥30 kg/m^2^) were also carried out. We found that CI had the best discrimination power among participants with a BMI <30 kg/m^2^ and participants with a BMI≥30 kg/m^2^. All of the eight anthropometric measures showed higher AUC values in the participants with a BMI <30 kg/m^2^ (Supplementary Table S3).

### Combination of BMI and other anthropometric indices

**Figure 4:**
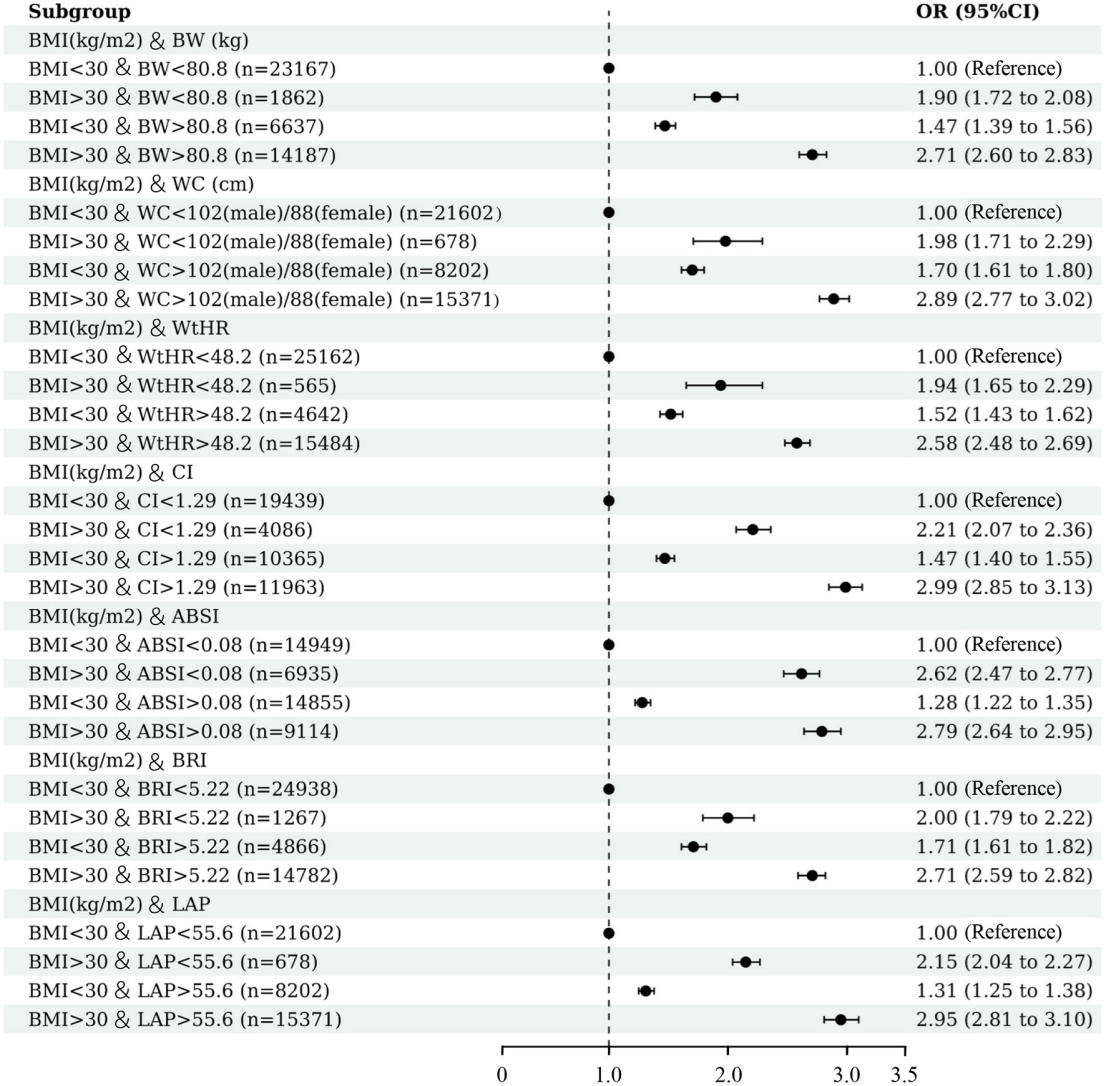
Association between hypertension and combined anthropometric indices. Multivariable logistic regression was conducted after adjusting for confounding factors of age, sex, race/ethnicity, education, smoking, drinking, diabetes, and eGFR.

Correlation analysis based on Spearman method was perform to evaluate the association between different anthropometric indices. Results of correlation analysis between different anthropometric indices show that body weight and BMI have the strongest correlation (*r* = 0.97, *P* < 0.001), and the correlation between ABSI and body weight is minimal (*r* = 0.1, *P* < 0.001) (Supplementary Figure S1). BMI is currently the most widely used of all body measures, so we combined BMI with other body measures to assess the risk of hypertension in this study. As shown in Figure 4, in individuals with BMI < 30 kg/m^2^, elevated body weight (OR 1.47; 95% CI: 1.39–1.56; *P* < 0.001), waist circumference (OR 1.70; 95% CI: 1.61–1.80; *P* < 0.001), WtHR (OR 1.52; 95% CI: 1.43–1.62; *P* < 0.001), CI (OR 1.47; 95% CI: 1.40–1.55; P <0.001), ABSI (OR 1.28; 95% CI: 1.22–1.35; *P* < 0.001), BRI (OR 1.71; 95% CI: 1.61–1.82; *P* < 0.001), LAP (OR 1.31; 95% CI: 1.25–1.38; *P* < 0.001) were all positively correlated with the occurrence of hypertension. In participants with normal body weight, waist circumference, WtHR, CI, ASBI, BRI and LAP, an elevated BMI also increased hypertension risk (all *P* < 0.001). Increments in both BMI and other anthropometric indices greatly increased the risk of hypertension. The ORs for body weight, waist circumference, WtHR, CI, ABSI, BRI, and LAP were 2.71 (95%CI: 2.60–2.83), 2.89 (95%CI: 2.77–3.02), 2.58 (95%CI: 2.60–2.83), 2.99 (95%CI: 2.85–3.13), 2.79 (95%CI: 2.64–2.95), 2.71 (95%CI: 2.59–2.82) and 2.95 (95%CI: 2.81–3.10), respectively.

### Nomogram model

**Figure 5:**
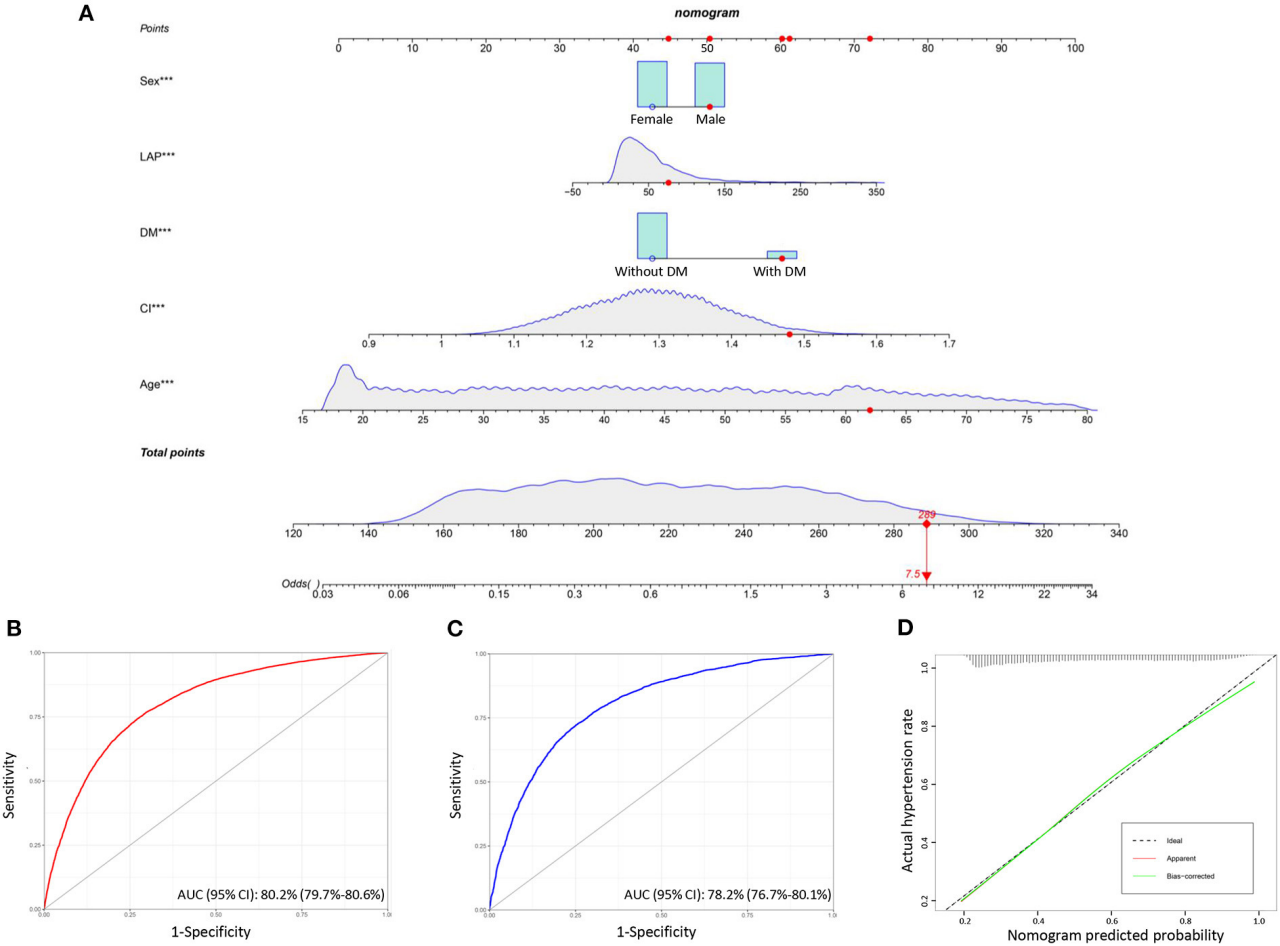
Nomogram model for predicting hypertension risk. **(A)** Nomogram model shows the distribution of risk factors included, correspond to the points at the top, and the sum of the scores of all variables is total points. Red points show an example, for a 62 years-old with diabetes, a CI of 1.44 and a LAP of 76, the probability of hypertension increased by 7.5-fold; **(B)** ROC curve of the nomogram model in the training group; **(C)** ROC curve of the nomogram model in the validation group; **(D)** calibration curve of the nomogram model based on bootstrap validation with 1,000 re-samplings in the training group.

We adopted nomogram model to further investigate the discriminatory power of novel anthropometric indices in screening hypertension. Considering most of anthropometric indices were calculated through combing body weight, waist circumference and Body, the inclusion of all indices in the regression model causes confusion in the model and reduces the diagnostic efficiency. Therefore, we selected CI (the most powerful measurement in ROC analysis of a single index) and LAP (the only measurement of lipid levels was introduced) to establish the regression model, the other confounding factors including, age, sex, race, education levels, and diabetes, were also included. All of the participants included in the present study were randomly were randomly assigned to the training group and the validation group in a ratio of 3 to 1. The nomogram model was firstly generated in the training group, after evaluation, we finally selected the variables with significant contributions (age, sex, diabetes, CI and LAP) to build the model. For example, for a 62 years-old with diabetes, a CI of 1.44 and a LAP of 76, the probability of hypertension increased by 7.5-fold (Figure 5A). ROC curves were used to evaluate the predicting ability of hypertension risk. In both training group and validation group, nomogram model showed superior predicting ability than single anthropometric measurement. The AUC (95% CI) in training group was 80.2% (79.7%-80.6%), the AUC (95% CI) in validation group was 79.5% (78.3%-80.1%), and there was no difference among them (Figures 5B,C). A calibration plot of by bootstrap validation with 1,000 re-samplings in the training group was also used to evaluate the consistency of model. Results of calibration plot demonstrated the nomogram model was well-constructed with good consistency and can be applied to predict the risk of hypertension (Figure 5D).

### Sensitivity analysis

Considering the application of medication could have an impact on the blood pressure of participants, individuals who received any drug treatment were excluded in sensitive analysis. The detailed baseline characteristics of participants included in the sensitive analysis were shown in Supplementary Table S4. The results of sensitive analysis were consistent with the main analysis. All of the eight anthropometric indices that were positively correlated with the prevalence of hypertension, BRI, WtHR and BMI had higher ORs as well (Supplementary Table S5). Likewise, restricted cubic splines of sensitive analysis showed that the association between different anthropometric indices and hypertension in a non-linear pattern. The trends of the restricted cubic splines were similar to the results in the main analysis (Supplementary Figure S2). In addition, CI had the best discrimination ability among the eight anthropometric parameters (AUC 0.659; 95% CI: 0.651–0.667; optimal cutoff value: 1.3). However, there was no the difference between the diagnostic abilities of BRI (AUC 0.65; 95% CI: 0.642–0.658; optimal cutoff value: 4.5) and CI in the sensitive analysis (Supplementary Figure S3).

## Discussion

Both abdominal obesity and hypertension are important components of metabolic syndrome, and their common pathogenesis includes insulin resistance, hyperinsulinemia and inflammation, which can cause damage to heart, kidney and other organs (17–19). Investigators has already focused on the important role of abdominal obesity in the occurrence of hypertension. Taleb et al. recently conducted a cross-sectional study investigated the correlation between BMI and waist circumference with blood pressure among 785 Algerian adults. Results of multivariable logistic regression indicated obesity increased the risk of hypertension by a factor of 1.54 [95% CI (1.15, 2.06)] (*p* = 0.004) (20). In another largescale cross-sectional study focused on the association between waist circumference and hypertension, Sun et al. found that waist circumference could be a novel index in evaluating risk of hypertension. Similar to the results of our study, they also demonstrated the non-linear association between increased waist circumference and risk of hypertension based on restricted cubic spline. However, they did not directly mention the discriminatory of waist circumference and BMI, and the novel anthropometric indices proposed recent years was not included either (14). The association between hypertension and gain in waist circumference was also investigated in a cohort study from rural China. 2,027 hypertension cases occurred in 10,265 participants (without hypertension) after a 6-year follow-up. They showed that the risk of hypertension was strongly increased in participants with waist circumference gain >5% compared to the reference group (−2.5% <waist circumference gain <2.5%) (21). Comparing with the measurements of general obesity (body weight and BMI), five abdominal obesity indices enrolled in the present study, including waist circumference, WtHR, ABSI, CI and BRI, had better discriminatory power in screening hypertension risk, reconfirming that abdominal obesity is more harmful for the prevalence of hypertension than general obesity.

Most studies used body weight and BMI as the main criteria for assessing obesity at present. However, body weight and BMI does not accurately reflect the distribution of body fat (22–24). In recent years, several novel anthropometric measurements were newly proposed to more accurately evaluate different obesity patterns and assess the relationship between obesity and development of chronic diseases. BRI, one of the novel anthropometric indices, was firstly created in 2013 to predict the percentage of visceral adipose, and then found to be superior to traditional anthropometric measurements in predicting various metabolic related diseases (25). A detailed meta-analysis revealed that AUCs for BRI predicting metabolic syndrome was higher than body weight, BMI, WtHR and ABSI. However, most of the original researches included in this meta-analysis were from China, whether the conclusion can be applied to other population remains unclear (26). Moreover, results from a recent observational study showed that BRI closely associated with hypertension-mediated organ damages, including lower limb atherosclerosis, microalbuminuria, arterial stiffness and left ventricular hypertrophy. They also discerned that BRI had a greater AUC in the detecting of hypertension related complications compared with BMI in female individuals but not male individuals (27). Similarly, due to limited race/age distribution and relatively small sample size (*n* = 3,337), the relationship between BRI and hypertension risk, as well as the diagnostic power of BRI need to be further explored. ABSI was firstly revealed as an independent predictor for all-cause mortality (28). A cross-sectional study enrolled 3,213 Iranian adults and investigated the relationship between psychological disorders and ABSI. Results showed that depression, anxiety and psychological distress were strongly associated with ABSI among female participants (29). ABSI has been shown to be positively associated with visceral fat and cardiovascular disease. In a cross-sectional study involving 607 patients with type 2 diabetes mellitus (mean age 64 ± 12 years, 60.0% male), visceral fat area, subcutaneous fat area, and brachial-ankle pulse wave velocity were measured. The results of this study showed that ABSI was positively correlated with visceral fat and could be used as a substitute for arterial stiffness in patients with T2DM (30). Moreover, evidence has already illustrated the predictive value of ABSI for risks of cardiovascular mortality and all-cause mortality (31). CI was also applied in the fields of predicting hypertension risk, Ghosh et al. adopted both linear regression analysis and logistic regression analysis to evaluate the specific relationship between CI and hypertension among 197 Indian girls aged 5–16 years. The investigators demonstrated that elevated CI was associated with an ~1.85-fold increased risk of hypertension (95% CI, 1.14–3.0) (32). In Western populations, CI has also been demonstrated as a sensitive indicator of hypertension. Mantzoros et al. enrolled 280 healthy women between 18 and 24 years old and found that CI was not only associated with fasting blood insulin levels and lipid profiles, but also a predictor for hypertension risk. CI was already found to be correlated with insulin resistance and dyslipidemia, the risk factors for arterial stiffness and hypertension (33). Hypertension, insulin resistance, and dyslipidemia are all influenced by visceral fat (34). Visceral fat has also been associated with increased activity of sympathetic nervous system, one of the most important causes of hypertension (35). Instead of indicating heavier body weight, LAP may better reflect the accumulation of total fat in the body and the function of visceral fat (36). Results from our study demonstrated that all of the eight anthropometric measurements (body weight, BMI, waist circumference, WtHR, CI, ABSI, BRI, and LAP) positively correlated with the prevalence of hypertension, supporting that weight losing plays an important role in the management of hypertension. Of note, novel anthropometric measurements had a stronger correlation with hypertension risk and a better performance in discriminating hypertension. For example, increased ABSI (per SD) had the strongest association with hypertension risk the best discriminatory ability. Therefore, focusing on roles of anthropometric indices, especially novel anthropometric indices, is helpful for the prevention, screening and treatment of hypertension. A nomogram model based on age, sex, diabetes, CI and LAP was also generated in the present study, which showed a favorable discriminating power and good consistency. Although this prediction cannot be applied to clinical practice at present, it provides a new idea for predicting the risk of hypertension through anthropometric indices.

In this study, subgroup analysis stratified by age, sex and BMI was also carried out. The age-related difference in hypertension risk has already been reported in several studies, which may be due to differences in the prevalence of hypertension in younger and older populations (37–39). Seok et al. reported that both increased BMI and waist circumference were strongly associated with hypertension risk. Similar to our results, across all BMI and waist circumference categories, the correlation was stronger in younger participants (19–39 years old) than those 40–64 years or >65 years (37). The incidence of hypertension is higher in the elderly population than in the young population, the degradation of vascular elasticity and peripheral vascular resistance caused by aging are important reasons (40–42). Hypertension in young people appears to be attributed more to the effects of a genetic background, unhealthy lifestyle habits and diverse comorbidities. With the increasing incidence of hypertension in young people, it is necessary to apply the new body measurement index in the prevention and treatment of hypertension (43). We also observed that the association between anthropometric indices and hypertension risk was stronger in individuals with <30 kg/m^2^ than in individuals with BMI ≥30 kg/m^2^. BMI, waist circumference, WtHR, CI, ABSI and BRI were more strongly correlated with hypertension in male individuals comparing with female individuals. However, LAP was more closely associated with hypertension risk in female individuals.

Restricted cubic spline, a widely used method, is applied to fit and analyze the non-linear relationship between variables and outcomes (44–46). When using restricted cubic spline to draw curve relation, it is usually necessary to set the number of knots. In most cases, the position of the knots has little influence on the fitting of the restricted cubic spline, while the number of knots determines the shape of the curve, or the degree of smoothness, and most researchers recommend 3 knots (47). Xu et al. adopted restricted cubic spline to investigate the non-linear (U-shape) association between physical activity and cardiocerebrovascular mortality. The results suggest that highly active physical activity (7.5–15 h/week) was associated with the lowest risks of cardiocerebrovascular and all-cause mortality in patients with hypertension (48). Another study used restricted cubic spline and explored the association between excessive dietary branched-chain amino acids (BCAAs) intake and hypertension risk in Chinese population, also indicating the non-linear positive relationship between BCAAs intake and the occurrence of hypertension. In the present study, restricted cubic splines (3 knots) were used to flexibly models and visualize the relationships of anthropometric indices with the risk of hypertension (49). Except for LAP and ABSI, the trends of the other six restricted cubic splines are similar, with the increase of anthropometric measurements, the risk of hypertension increased more rapidly. Therefore, the results of logistic regression analyses and restricted cubic spline analyses both indicated that early attention to anthropometric indices is crucial to prevent the occurrence of hypertension.

There are several advantages and implications of our study. First, it was adequate to provide reliable conclusion considering the large-scale sample size included; second, our study adopted restricted cubic splines and further demonstrated the non-linear associations between hypertension risk and anthropometric measurements, the trends of restricted cubic splines and cutoff values may provide help for health policy makers; third, results of our present study demonstrated again that abdominal obesity have a strong association with hypertension and abdominal obesity indices showed a superior discriminating effectiveness in screening the risk of hypertension than traditional anthropometric measurements; fourth, we used the method of “multiple imputation” to fill missing covariates, which avoids the selection bias due to excluding participants with missing data. However, several limitations of this study also have to be clarified. First, the causal associations could not be determined considering the research type of cross-sectional study, more prospective studies are needed to determine the exact relationship between different forms of obesity and hypertension; second, there may exist experienced subjective bias due to the self-reported covariates from NHANES database; third, there are large ethnic differences in diet, physical activity, genetic variants, lipid metabolism, and susceptibility to cardiovascular disease. Therefore, whether the conclusion in the present study based on US participants could be applicable to other populations need to be further explored in the future work.

## Conclusion

We retrospectively analyzed 45,853 adults in the US from NHANES and found that anthropometric measurements, including body weight, BMI, waist circumference, WtHR, CI, ABSI, BRI, and LAP closely associated with hypertension risk. Among all anthropometric indices, ABSI (per SD increment) had the strongest positive association with hypertension, CI had superior discriminatory power for detecting hypertension risk. BMI alone is insufficient for evaluating the risk of hypertension. More attention should be paid to anthropometric indices, especially novel anthropometric indices, to better prevent and treat of hypertension.

## Data availability statement

The original contributions presented in the study are included in the article/Supplementary material, further inquiries can be directed to the corresponding author/s.

## Ethics statement

The studies involving human participants were reviewed and approved by National Center for Health Statistics Research Ethics Review Board. The participants provided informed consent to participate in the NHANES survey. The patients/participants provided their written informed consent to participate in this study.

## Author contributions

S-LC and J-XZ were involved in the experiment design. L-DW and YS performed the data analysis. L-DW and C-HK wrote the manuscript. C-HK and YS reviewed the manuscript and provided critical suggestions. All authors contributed to the article and approved the submitted version.

## Funding

This research was funded by the National Natural Science Foundation of China (Grant Nos. 81700398, 81970309, and 81770441) and the Natural Science Foundation of Jiangsu Province (Grant No. BK20191117).

## Conflict of interest

The authors declare that the research was conducted in the absence of any commercial or financial relationships that could be construed as a potential conflict of interest.

## Publisher's note

All claims expressed in this article are solely those of the authors and do not necessarily represent those of their affiliated organizations, or those of the publisher, the editors and the reviewers. Any product that may be evaluated in this article, or claim that may be made by its manufacturer, is not guaranteed or endorsed by the publisher.

## Supplementary material

Supplementary Figure S1 Sperman correlation analysis among anthropometric measurements. Numbers represent correlation coefficients. The darker the red indicates the stronger the positive correlation, the darker the blue indicates the stronger the negative correlation. BW, body weight; BMI, body mass index; WC, waist circumference; WtHR, waist-to-height ratio; CI, conicity index; ABSI, a body shape index; BRI, body round index; LAP, lipid accumulation product.

Supplementary Figure S2 Distribution of the anthropometric measurements of participants (without medical therapy) enrolled in sensitive analysis and restricted cubic splines. The distribution histogram is represented in the background and restricted cubic spline analyses were adjusted for age, sex, race/ethnicity, education, smoking, drinking, diabetes, and eGFR.

Supplementary Figure S3 ROC curves of anthropometric indices for discriminating hypertension in sensitive analysis. ROC, receiver operating characteristic; AUC, area under the curve; BW, body weight; BMI, body mass index; WC, waist circumference; WtHR, waist-to-height ratio; CI, conicity index; ABSI, a body shape index; BRI, body round index; LAP, lipid accumulation product.

Supplementary Table S1 Baseline characteristics grouped by sex.

Supplementary Table S2 Subgroups analysis stratified by age, sex, BMI, and race.

Supplementary Table S3 ROC analyses in subgroups stratified by BMI.

Supplementary Table S4 Comparison of baseline characteristics among participants without any medical therapy.

Supplementary Table S5 Sensitivity analysis of anthropometric measures and hypertension among participants without any medical therapy.
