# Supplementary figures and images for: Associations between novel anthropometric measures and the prevalence of hypertension among 45,853 adults: A cross-sectional study
Source: Front Cardiovasc Med. 2022 Nov 3;9:1050654. doi: 10.3389/fcvm.2022.1050654 (PMC9669705; doi:10.3389/fcvm.2022.1050654)

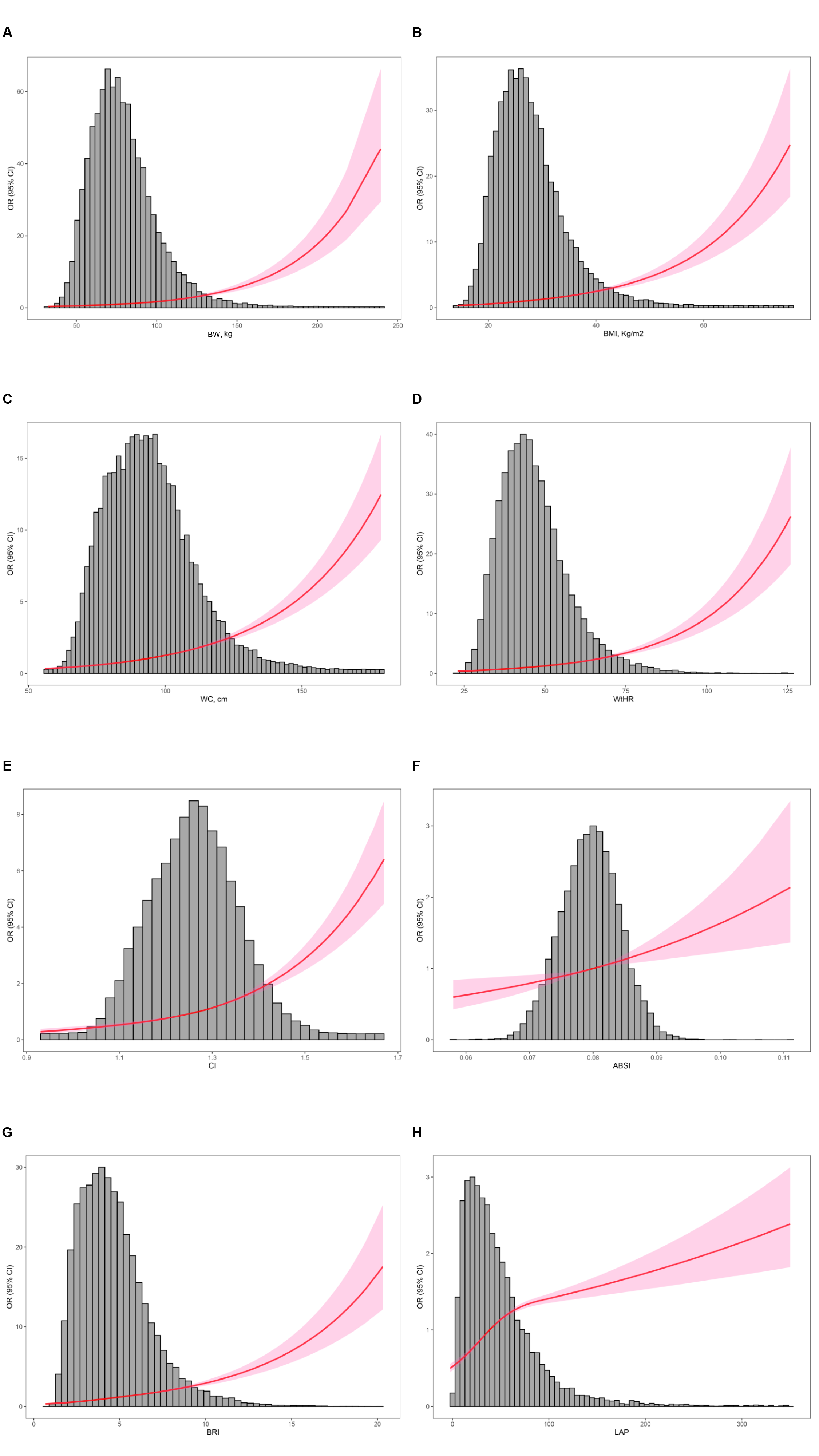

Supplement: Supplementary Figure S1 — Sperman correlation analysis among anthropometric measurements. Numbers represent correlation coefficients. The darker the red indicates the stronger the positive correlation, the darker the blue indicates the stronger the negative correlation. BW, body weight; BMI, body mass index; WC, waist circumference; WtHR, waist-to-height ratio; CI, conicity index; ABSI, a body shape index; BRI, body round index; LAP, lipid accumulation product. [file Image_1.TIF]

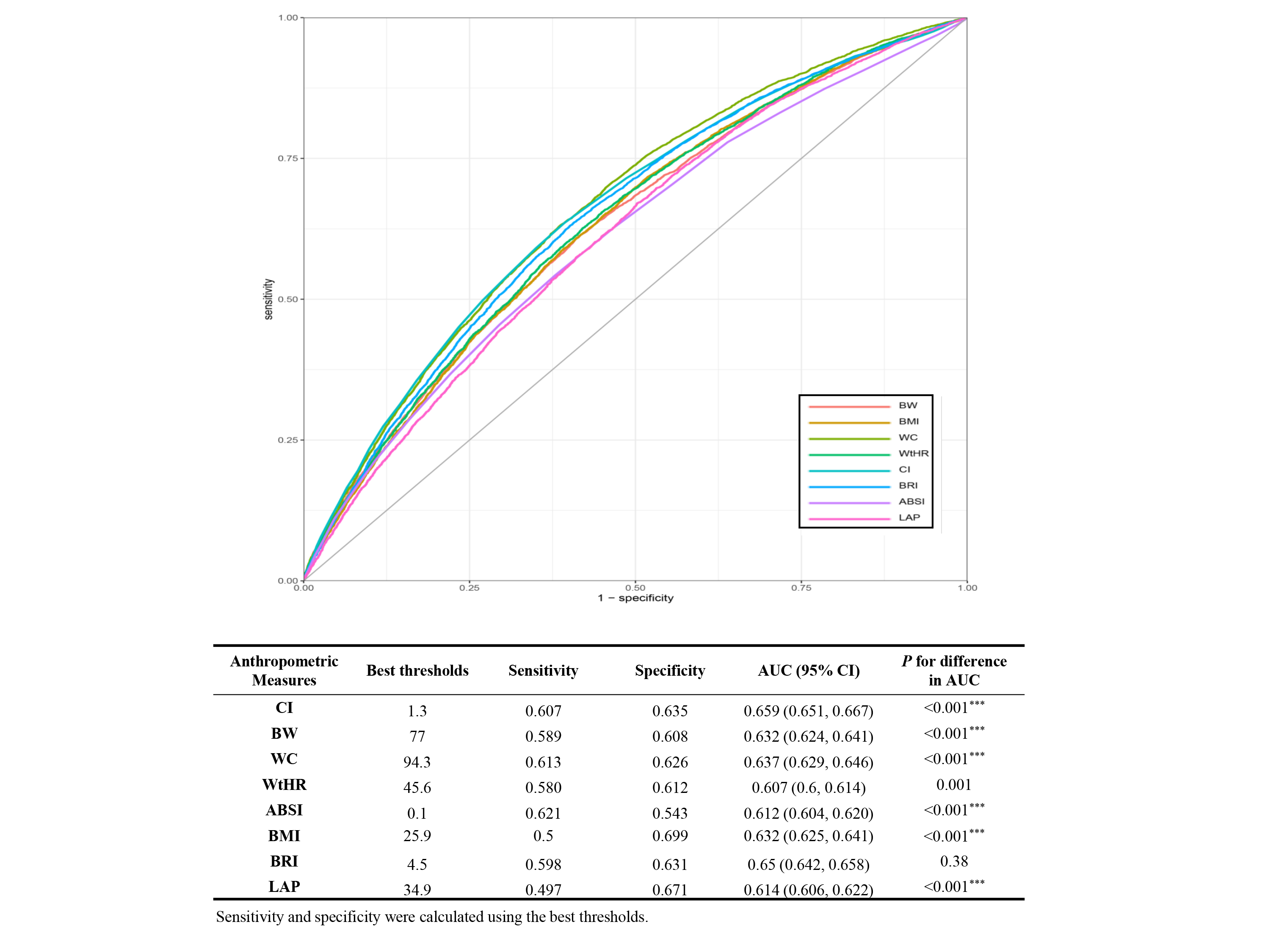

Supplement: Supplementary Figure S2 — Distribution of the anthropometric measurements of participants (without medical therapy) enrolled in sensitive analysis and restricted cubic splines. The distribution histogram is represented in the background and restricted cubic spline analyses were adjusted for age, sex, race/ethnicity, education, smoking, drinking, diabetes, and eGFR. [file Image_2.TIF]

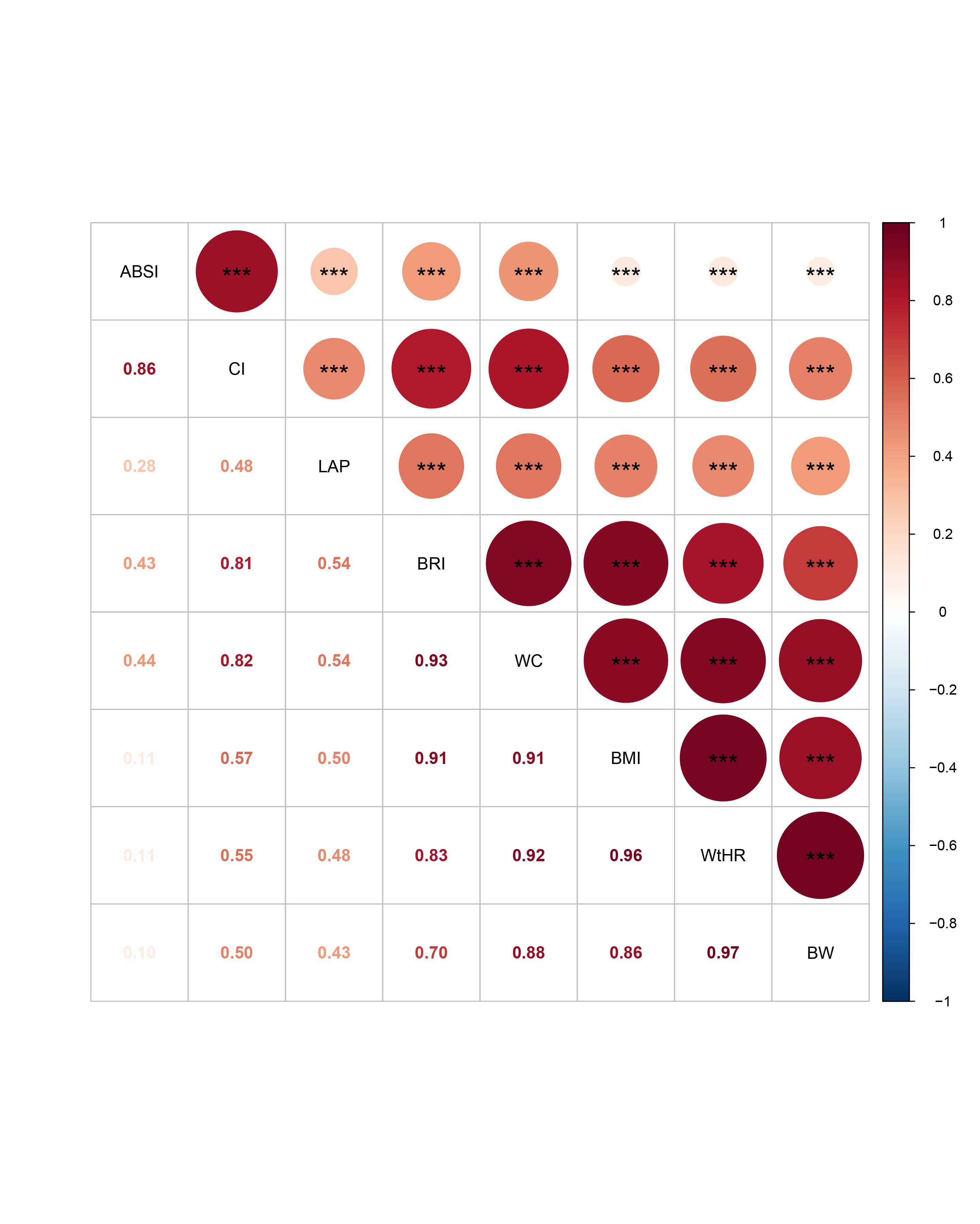

Supplement: Supplementary Figure S3 — ROC curves of anthropometric indices for discriminating hypertension in sensitive analysis. ROC, receiver operating characteristic; AUC, area under the curve; BW, body weight; BMI, body mass index; WC, waist circumference; WtHR, waist-to-height ratio; CI, conicity index; ABSI, a body shape index; BRI, body round index; LAP, lipid accumulation product. [file Image_3.TIF]
