# Supplementary material for: Associations between novel anthropometric measures and the prevalence of hypertension among 45,853 adults: A cross-sectional study
Source: Front Cardiovasc Med. 2022 Nov 3;9:1050654. doi: 10.3389/fcvm.2022.1050654 (PMC9669705; doi:10.3389/fcvm.2022.1050654)
Supplement: Supplementary Table S1 — Baseline characteristics grouped by sex. [file Table_1.DOCX]

**Table S1.** **Baseline Characteristics Grouped by Sex.**

| Variables | Male | | | | Female | | | | *P* value between male and female |
| --- | --- | --- | --- | --- | --- | --- | --- | --- | --- |
|  | **Overall**  **(n = 22962)** | **Non-hypertension (n = 12387)** | **Hypertension (n = 10575)** | ***P* value** | **Overall**  **(n = 22891)** | **Non-hypertension (n = 13564)** | **Hypertension (n = 9327)** | ***P* value** |  |
| Age, years | 44.05 ± 17.27 | 37.12 ± 15.38 | 52.16 ± 15.76 | <0.001*** | 44.38 ± 16.89 | 36.91 ± 14.46 | 55.23 ± 14.04 | <0.001*** | 0.32 |
| Race, n (%) |  |  |  | <0.001*** |  |  |  | <0.001*** | <0.001*** |
| Non-Hispanic White | 9400 (40.9) | 4935 (39.8) | 4465 (42.2) |  | 8972 (39.2) | 5416 (39.9) | 3556 (38.1) |  |  |
| Non-Hispanic Black | 5024 (21.9) | 2322 (18.7) | 2702 (25.6) |  | 5096 (22.3) | 2490 (18.4) | 2606 (27.9) |  |  |
| Mexican American | 4503 (19.6) | 2745 (22.2) | 1758 (16.6) |  | 4450 (19.4) | 2859 (21.1) | 1591 (17.1) |  |  |
| Other Hispanic | 1797 (7.8) | 1040 (8.4) | 757 (7.2) |  | 2161 (9.4) | 1345 (9.9) | 816 (8.7) |  |  |
| Other | 2238 (9.7) | 1345 (10.9) | 893 (8.4) |  | 2212 (9.7) | 1454 (10.7) | 758 (8.1) |  |  |
| Smoking, n (%) | 12160 (53.0) | 5970 (48.2) | 6190 (58.5) | <0.001*** | 8047 (35.2) | 4364 (32.2) | 3683 (39.5) | <0.001*** | <0.001*** |
| Drinking, n (%) | 15282 (66.6) | 8113 (65.5) | 7169 (67.8) | <0.001*** | 12666 (55.3) | 7767 (57.3) | 4899 (52.5) | <0.001*** | <0.001*** |
| Education level, n (%) |  |  |  | <0.001*** |  |  |  | <0.001*** | <0.001*** |
| Below high school | 6565 (28.6) | 3508 (28.3) | 3057 (28.9) |  | 5882 (25.7) | 3120 (23.0) | 2762 (29.6) |  |  |
| High school | 5683 (24.7) | 3061 (24.7) | 2622 (24.8) |  | 5269 (23.0) | 3009 (22.2) | 2260 (24.2) |  |  |
| Above high school | 10714 (46.7) | 5818 (47.0) | 4896 (46.3) |  | 11740 (51.3) | 7435 (54.8) | 4305 (46.2) |  |  |
| SBP, mmHg | 123.73 ± 16.07 | 114.77 ± 8.38 | 134.22 ± 16.58 | <0.001*** | 120.19 ± 18.81 | 110.07 ± 9.47 | 134.91 ± 19.29 | <0.001*** | <0.001*** |
| DBP, mmHg | 72.03 ± 12.11 | 68.05 ± 9.54 | 76.70 ± 13.09 | <0.001*** | 69.79 ± 11.15 | 66.79 ± 8.76 | 74.15 ± 12.70 | <0.001*** | <0.001*** |
| Diabetes, n (%) | 3294 (14.3) | 832 (6.7) | 2462 (23.3) | <0.001*** | 3096 (13.5) | 768 (5.7) | 2328 (25.0) | <0.001*** | 0.36 |
| FBG, mmol/L | 5.97 ± 1.95 | 5.69 ± 1.61 | 6.31 ± 2.24 | <0.001*** | 5.77 ± 1.84 | 5.45 ± 1.33 | 6.23 ± 2.33 | <0.001*** | <0.001*** |
| HbA1c, % | 5.66 ± 1.06 | 5.47 ± 0.88 | 5.89 ± 1.21 | <0.001*** | 5.62 ± 1.03 | 5.39 ± 0.76 | 5.96 ± 1.26 | <0.001*** | <0.001*** |
| eGFR, ml/min/1.73m2 | 99.23 ± 19.02 | 104.53 ± 18.41 | 93.02 ± 17.80 | <0.001*** | 102.13 ± 20.17 | 108.31 ± 19.10 | 93.14 ± 18.20 | <0.001*** | <0.001*** |
| Anthropometric measures |  |  |  |  |  |  |  |  |  |
| BW, kg | 86.13 ± 20.28 | 81.92 ± 17.95 | 91.06 ± 21.69 | <0.001*** | 75.5 ± 20.43 | 71.67 ± 18.56 | 81.08 ± 21.71 | <0.001*** | <0.001*** |
| BMI, kg/m2 | 28.2 ± 5.96 | 26.81 ± 5.27 | 29.82 ± 6.30 | <0.001*** | 29.13 ± 7.45 | 27.48 ± 6.79 | 31.52 ± 7.71 | <0.001*** | <0.001*** |
| WC, cm | 99.06 ± 16.02 | 94.26 ± 14.51 | 104.67 ± 15.89 | <0.001*** | 95.61 ± 16.79 | 91.10 ± 15.52 | 102.17 ± 16.39 | <0.001*** | <0.001*** |
| WtHR | 49.23 ± 10.79 | 46.82 ± 9.51 | 52.06 ± 11.48 | <0.001*** | 46.85 ± 12.16 | 44.33 ± 11.05 | 50.51 ± 12.75 | <0.001*** | <0.001*** |
| CI | 1.30 ± 0.09 | 1.26 ± 0.09 | 1.33 ± 0.09 | <0.001*** | 1.29 ± 0.09 | 1.26 ± 0.09 | 1.32 ± 0.09 | <0.001*** | <0.001*** |
| ABSI | 0.09 ± 0.003 | 0.08 ± 0.003 | 0.09 ± 0.004 | <0.001*** | 0.08 ± 0.003 | 0.08 ± 0.002 | 0.08 ± 0.01 | <0.001*** | <0.001*** |
| BRI | 4.89 ± 2.05 | 4.29 ± 1.78 | 5.60 ± 2.12 | <0.001*** | 5.54 ± 2.51 | 4.85 ± 2.23 | 6.54 ± 2.55 | <0.001*** | <0.001*** |
| LAP | 55.06 ± 4.84 | 47.87 ± 4.33 | 63.48 ± 5.25 | <0.001*** | 55.06 ± 4.4 | 47.54 ± 4.01 | 65.99 ± 4.71 | <0.001*** | 0.994 |
| TG, mmol/L | 1.55 ± 1.32 | 1.51 ± 1.20 | 1.60 ± 1.44 | <0.001*** | 1.41 ± 1.07 | 1.34 ± 1.05 | 1.50 ± 1.09 | <0.001*** | <0.001*** |
| TC, mmol/L | 4.97 ± 1.10 | 4.87 ± 1.06 | 5.08 ± 1.13 | <0.001*** | 5.05 ± 1.06 | 4.89 ± 1.02 | 5.28 ± 1.08 | <0.001*** | <0.001*** |
| LDL-C, mmol/L | 3.02 ± 1.02 | 2.93 ± 0.99 | 3.12 ± 1.04 | <0.001*** | 2.93 ± 0.98 | 2.80 ± 0.94 | 3.13 ± 1.00 | <0.001*** | <0.001*** |
| HDL-C, mmol/L | 1.24 ± 0.36 | 1.25 ± 0.34 | 1.24 ± 0.38 | 0.078 | 1.47 ± 0.41 | 1.48 ± 0.40 | 1.46 ± 0.43 | 0.057 | <0.001*** |
| RBC, ×109/L | 5.00 ± 0.44 | 5.03 ± 0.42 | 4.96 ± 0.47 | <0.001*** | 4.49 ± 0.39 | 4.47 ± 0.37 | 4.51 ± 0.41 | <0.001*** | <0.001*** |
| WBC, ×109/L | 7.12 ± 2.22 | 7.01 ± 2.13 | 7.24 ± 2.32 | <0.001*** | 7.27 ± 2.20 | 7.21 ± 2.08 | 7.35 ± 2.36 | <0.001*** | <0.001*** |
| NE, ×109/L | 4.15 ± 1.65 | 4.06 ± 1.63 | 4.26 ± 1.66 | <0.001*** | 4.26 ± 1.68 | 4.25 ± 1.66 | 4.28 ± 1.72 | 0.154 | <0.001*** |
| Monocyte, ×109/L | 0.58 ± 0.20 | 0.56 ± 0.19 | 0.59 ± 0.20 | <0.001*** | 0.52 ± 0.18 | 0.52 ± 0.17 | 0.53 ± 0.18 | <0.001*** | <0.001*** |
| LY, ×109/L | 2.13 ± 1.05 | 2.13 ± 0.90 | 2.13 ± 1.20 | 0.74 | 2.25 ± 0.90 | 2.22 ± 0.68 | 2.29 ± 1.15 | <0.001*** | <0.001*** |
| PLT, ×106/L | 240.08 ± 59.09 | 241.63 ± 57.74 | 238.27 ± 60.59 | <0.001*** | 271.13 ± 69.2 | 270.24 ± 67.31 | 272.42 ± 71.83 | 0.019* | <0.001*** |
| Hemoglobin, g/L | 15.17 ± 1.18 | 15.26 ± 1.09 | 15.06 ± 1.27 | <0.001*** | 13.36 ± 1.23 | 13.32 ± 1.19 | 13.41 ± 1.27 | <0.001*** | <0.001*** |

Variables are presented as the mean ± SD (continuous) or number with percent (categorical). SD, standard deviation; SBP, systolic blood pressure; DBP, diastolic blood pressure; FBG, fasting blood glucose; HbA1c, glycated hemoglobin; eGFR, estimated glomerular filtration rate; BW, body weight; BMI, body mass index; WC, waist circumference; WtHR, waist-to-height ratio; CI, conicity index; ABSI, a body shape index; BRI, body round index; LAP, lipid accumulation product; TG, triglycerides; TC, total cholesterol; LDL-C, low-density lipoprotein cholesterol; HDL-C, high-density lipoprotein cholesterol; RBC, red blood cells; WBC, white blood cells; NE, neutrophils; LY, lymphocytes; PLT, platelets. *** *P* value<0.001, ** *P* value<0.01, * *P* value<0.05.
