# Supplementary material for: Associations between novel anthropometric measures and the prevalence of hypertension among 45,853 adults: A cross-sectional study
Source: Front Cardiovasc Med. 2022 Nov 3;9:1050654. doi: 10.3389/fcvm.2022.1050654 (PMC9669705; doi:10.3389/fcvm.2022.1050654)
Supplement: Supplementary Table S2 — Subgroups analysis stratified by age, sex, BMI, and race. [file Table_2.DOCX]

**Table S2. Subgroups Analysis Stratified by Age, Sex, BMI, Race**

|  | Age | | Sex | | BMI | | Race | | |
| --- | --- | --- | --- | --- | --- | --- | --- | --- | --- |
|  | **<60 years (n=16148)** | **≥60 years (n=35290)** | **Males (n=25680)** | **Females (n=25758)** | **<30 kg/m^2^ (n=17929)** | **≥30 kg/m^2^ (n=33509)** | **White (n=21995)** | **Black (n=11060)** | **Other (n=18383)** |
| BW | 1.71 [1.68, 1.75] | 1.51 [1.43, 1.60] | 1.69 [1.64, 1.73] | 1.68 [1.63, 1.73] | 1.58 [1.50, 1.66] | 1.46 [1.41, 1.52] | 1.73 [1.68, 1.79] | 1.54 [1.48, 1.60] | 1.71 [1.65, 1.78] |
| *P* for interaction | <0.001^***^ | | 0.703 | | <0.001^***^ | | 0.02^*^ | 0.042^*^ | 0.008^**^ |
| BMI | 1.71 [1.67, 1.75] | 1.55 [1.47, 1.64] | 1.8 [1.74, 1.85] | 1.61 [1.57, 1.66] | \ | \ | 1.75 [1.69, 1.80] | 1.53 [1.48, 1.59] | 1.68 [1.62, 1.74] |
| *P* for interaction | <0.001^***^ | | <0.001^***^ | | \ | | 0.005^**^ | 0.78 | 0.58 |
| WC | 1.75 [1.71, 1.79] | 1.56 [1.48, 1.64] | 1.75 [1.70, 1.80] | 1.71 [1.66, 1.76] | 1.68 [1.60, 1.75] | 1.49 [1.43, 1.56] | 1.78 [1.72, 1.83] | 1.58 [1.52, 1.64] | 1.7 [1.64, 1.76] |
| *P* for interaction | <0.001^***^ | | 0.035^*^ | | <0.001^***^ | | 0.005^**^ | 0.87 | 0.93 |
| WtHR | 1.71 [1.68, 1.75] | 1.54 [1.46, 1.63] | 1.74 [1.69, 1.79] | 1.64 [1.59, 1.69] | 1.72 [1.63, 1.81] | 1.48 [1.43, 1.54] | 1.74 [1.69, 1.80] | 1.53 [1.48, 1.59] | 1.7 [1.64, 1.76] |
| *P* for interaction | <0.001^***^ | | 0.006^**^ | | <0.001^***^ | | <0.001^***^ | 0.12 | 0.13 |
| CI | 1.56 [1.53, 1.6] | 1.36 [1.29, 1.42] | 1.63 [1.58, 1.68] | 1.46 [1.42, 1.51] | 1.33 [1.29, 1.38] | 1.21 [1.16, 1.25] | 1.57 [1.52, 1.63] | 1.47 [1.41, 1.53] | 1.42 [1.37, 1.48] |
| *P* for interaction | <0.001^***^ | | 0.04^*^ | | <0.001^***^ | | <0.001^***^ | 0.96 | 0.003^**^ |
| ABSI | 4.21 [3.27, 5.42] | 2.51 [1.56, 4.04] | 4.67 [3.50, 7.18] | 2.14 [1.59, 2.88] | 4.90 [3.66, 6.55] | 1.41 [0.99, 2.01] | 4.59 [3.27, 6.43] | 5.17 [3.27, 8.18] | 1.29 [0.88, 1.9] |
| *P* for interaction | <0.001^***^ | | <0.001^***^ | | <0.001^***^ | | <0.001^***^ | 0.73 | 0.04^*^ |
| BRI | 1.74 [1.7, 1.78] | 1.55 [1.47, 1.64] | 1.83 [1.77, 1.89] | 1.64 [1.59, 1.69] | 1.89 [1.79, 1.99] | 1.39 [1.34, 1.44] | 1.78 [1.72, 1.84] | 1.58 [1.52, 1.65] | 1.65 [1.59, 1.71] |
| *P* for interaction | <0.001^***^ | | <0.001^***^ | | <0.001^***^ | | <0.001^***^ | 0.06 | 0.65 |
| LAP | 1.32 [1.29, 1.35] | 1.2 [1.15, 1.26] | 1.28 [1.24, 1.31] | 1.34 [1.3, 1.38] | 1.18 [1.15, 1.22] | 1.17 [1.13, 1.20] | 1.37 [1.33, 1.41] | 1.31 [1.25, 1.38] | 1.23 [1.20, 1.27] |
| *P* for interaction | <0.001^***^ | | <0.001^***^ | | <0.001^***^ | | <0.001^***^ | 0.09 | 0.002^**^ |

Data are presented as OR [95% CI], and *P* values for per SD increment. Age, sex, race/ethnicity, smoking, drinking, education levels, diabetes and eGFR were adjusted in subgroup analysis. OR, odds ratio; CI, confidence interval; SD, standard deviation; BW, body weight; BMI, body mass index; WC, waist circumference; WtHR, waist-to-height ratio; CI, conicity index; ABSI, a body shape index; BRI, body round index; LAP, lipid accumulation product. *** *P* value<0.001, ** *P* value<0.01, * *P* value<0.05.
