# Supplementary material for: Associations between novel anthropometric measures and the prevalence of hypertension among 45,853 adults: A cross-sectional study
Source: Front Cardiovasc Med. 2022 Nov 3;9:1050654. doi: 10.3389/fcvm.2022.1050654 (PMC9669705; doi:10.3389/fcvm.2022.1050654)
Supplement: Supplementary Table S3 — ROC analyses in subgroups stratified by BMI. [file Table_3.DOCX]

**Table S3. ROC Analyses in Subgroups Stratified by BMI**

| **Subgroups** | **Anthropometric**  **Measures** | **Best thresholds** | **Sensitivity** | **Specificity** | **AUC (95% CI)** | ***P* for difference**  **in AUC** |
| --- | --- | --- | --- | --- | --- | --- |
| **BMI<30 kg/m^2^** |  |  |  |  |  |  |
|  | **CI** | 1.3 | 0.675 | 0.635 | 0.707 (0.701, 0.713) | Reference |
|  | **BW** | 70.2 | 0.556 | 0.565 | 0.585 (0.578, 0.592) | <0.001^***^ |
|  | **BMI** | 25.2 | 0.588 | 0.599 | 0.621 (0.614, 0.627) | <0.001^***^ |
|  | **WC** | 90 | 0.633 | 0.640 | 0.684 (0.678, 0.691) | <0.001^***^ |
|  | **WtHR** | 41.7 | 0.548 | 0.612 | 0.607 (0.6, 0.614) | <0.001^***^ |
|  | **ABSI** | 0.1 | 0.678 | 0.598 | 0.687 (0.681, 0.693) | <0.001^***^ |
|  | **BRI** | 4.0 | 0.603 | 0.671 | 0.687 (0.681, 0.693) | <0.001^***^ |
|  | **LAP** | 28.2 | 0.462 | 0.694 | 0.604 (0.598, 0.611) | <0.001^***^ |
| **BMI≥30 kg/m^2^** |  |  |  |  |  |  |
|  | **CI** | 1.4 | 0.635 | 0.551 | 0.628 (0.619, 0.636) | Reference |
|  | **BW** | 101.5 | 0.642 | 0.444 | 0.555 (0.546, 0.564) | <0.001^***^ |
|  | **BMI** | 33.9 | 0.527 | 0.341 | 0.557 (0.549, 0.566) | <0.001^***^ |
|  | **WC** | 109.2 | 0.51 | 0.657 | 0.619 (0.667, 0.692) | <0.01^***^ |
|  | **WtHR** | 57.5 | 0.547 | 0.544 | 0.560 (0.551, 0.569) | <0.001^***^ |
|  | **ABSI** | 0.1 | 0.619 | 0.543 | 0.611 (0.602, 0.619) | <0.001^***^ |
|  | **BRI** | 6.6 | 0.468 | 0.702 | 0.61 (0.601, 0.618) | <0.001^***^ |
|  | **LAP** | 54.4 | 0.475 | 0.635 | 0.574 (0.566, 0.583) | <0.001^***^ |

Sensitivity and Specificity were calculated using the best thresholds. ROC, receiver operator characteristic curve; BW, body weight; BMI, body mass index; WC, waist circumference; WtHR, waist-to-height ratio; CI, conicity index; ABSI, a body shape index; BRI, body round index; LAP, lipid accumulation product. *** *P* value<0.001, ** *P* value<0.01, * *P* value<0.05.
