# Supplementary material for: Associations between novel anthropometric measures and the prevalence of hypertension among 45,853 adults: A cross-sectional study
Source: Front Cardiovasc Med. 2022 Nov 3;9:1050654. doi: 10.3389/fcvm.2022.1050654 (PMC9669705; doi:10.3389/fcvm.2022.1050654)
Supplement: Supplementary Table S4 — Comparison of baseline characteristics among participants without any medical therapy. [file Table_4.DOCX]

**Table S4. Comparison of Baseline Characteristics among Participants without any Medical Therapy**

| Variables | Overall  (n = 22817) | Non-hypertension  (n = 16739) | Hypertension  (n = 6078) | *P* value |
| --- | --- | --- | --- | --- |
| Age, years | 37.00 + 14.68 | 34.09 + 13.27 | 45.01 + 15.39 | <0.001*** |
| Sex-male, n (%) | 12976 (56.9) | 8994 (53.7) | 3982 (65.5) | <0.001*** |
| Race, n (%) |  |  |  | <0.001*** |
| Non-Hispanic White | 7305 (32.0) | 5383 (32.2) | 1922 (31.6) |  |
| Non-Hispanic Black | 5244 (23.0) | 3511 (21.0) | 1733 (28.5) |  |
| Mexican American | 5612 (24.6) | 4271 (25.5) | 1341 (22.1) |  |
| Other Hispanic | 2131 (9.3) | 1610 (9.6) | 521 (8.6) |  |
| Other | 2525 (11.1) | 1964 (11.7) | 561 (9.2) |  |
| Smoking, n (%) | 9102 (39.9) | 6222 (37.2) | 2880 (47.4) | <0.001*** |
| Drinking, n (%) | 8766 (38.4) | 6688 (40.0) | 2078 (34.2) | <0.001*** |
| Education level, n (%) |  |  |  | <0.001*** |
| Below high school | 6827 (29.9) | 4850 (29.0) | 1977 (32.5) |  |
| High school | 5524 (24.2) | 4000 (23.9) | 1524 (25.1) |  |
| Above high school | 10466 (45.9) | 7889 (47.1) | 2577 (42.4) |  |
| SBP, mmHg | 118.67 + 15.86 | 112.24 + 9.17 | 136.40 + 16.86 | <0.001*** |
| DBP, mmHg | 70.24 + 11.63 | 67.12 + 9.32 | 78.85 + 12.94 | <0.001*** |
| Diabetes, n (%) | 999 (4.4) | 471 (2.8) | 528 (8.7) | <0.001*** |
| FBG, mmol/L | 5.56 + 1.39 | 5.45 + 1.16 | 5.85 + 1.84 | <0.001*** |
| HbA1c, % | 5.42 + 0.77 | 5.35 + 0.65 | 5.63 + 1.02 | <0.001*** |
| eGFR, ml/min/1.73m2 | 107.06 + 18.66 | 109.44 + 18.25 | 100.53 + 18.24 | <0.001*** |
| Anthropometric measures |  |  |  |  |
| BW, kg | 78.86 + 20.19 | 76.31 + 18.73 | 85.87 + 22.28 | <0.001*** |
| BMI, kg/m2 | 27.68 + 6.36 | 26.91 + 5.97 | 29.79 + 6.90 | <0.001*** |
| WC, cm | 94.00 + 15.65 | 91.64 + 14.74 | 100.51 + 16.25 | <0.001*** |
| WtHR | 46.63 + 10.97 | 45.24 + 10.21 | 50.48 + 12.01 | <0.001*** |
| CI | 1.27 + 0.09 | 1.25 + 0.09 | 1.30 + 0.09 | <0.001*** |
| ABSI | 0.08 + 0.003 | 0.08 + 0.004 | 0.08 + 0.003 | <0.001*** |
| BRI | 4.71 + 2.12 | 4.42 + 1.97 | 5.49 + 2.31 | <0.001*** |
| LAP | 49.75 + 4.38 | 45.75 + 4.08 | 60.78 + 4.96 | <0.001*** |
| TG, mmol/L | 1.46 + 1.15 | 1.41 + 1.07 | 1.60 + 1.35 | <0.001*** |
| TC, mmol/L | 4.94 + 1.07 | 4.81 + 1.02 | 5.29 + 1.11 | <0.001*** |
| LDL-C, mmol/L | 2.94 + 1.00 | 2.83 + 0.97 | 3.25 + 1.03 | <0.001*** |
| HDL-C, mmol/L | 1.34 + 0.39 | 1.35 + 0.38 | 1.32 + 0.41 | <0.001*** |
| RBC, ×109/L | 4.81 + 0.49 | 4.79 + 0.49 | 4.88 + 0.49 | <0.001*** |
| WBC, ×109/L | 7.13 + 2.11 | 7.09 + 2.06 | 7.26 + 2.23 | <0.001*** |
| NE, ×109/L | 4.15 + 1.66 | 4.13 + 1.64 | 4.22 + 1.70 | <0.001*** |
| Monocyte, ×109/L | 0.55 + 0.18 | 0.54 + 0.18 | 0.56 + 0.19 | <0.001*** |
| LY, ×109/L | 2.19 + 0.77 | 2.18 + 0.69 | 2.23 + 0.96 | <0.001*** |
| PLT, ×106/L | 255.88 + 63.79 | 255.61 + 63.41 | 256.63 + 64.82 | <0.001*** |
| Hemoglobin, g/L | 14.43 + 1.55 | 14.35 + 1.54 | 14.65 + 1.56 | <0.001*** |

Variables are presented as the mean ± SD (continuous) or number with percent (categorical). SD, standard deviation; SBP, systolic blood pressure; DBP, diastolic blood pressure; FBG, fasting blood glucose; HbA1c, glycated hemoglobin; eGFR, estimated glomerular filtration rate; BW, body weight; BMI, body mass index; WC, waist circumference; WtHR, waist-to-height ratio; CI, conicity index; ABSI, a body shape index; BRI, body round index; LAP, lipid accumulation product; TG, triglycerides; TC, total cholesterol; LDL-C, low-density lipoprotein cholesterol; HDL-C, high-density lipoprotein cholesterol; RBC, red blood cells; WBC, white blood cells; NE, neutrophils; LY, lymphocytes; PLT, platelets. *** *P* value<0.001, ** P value<0.01, * *P* value<0.05.
