# Supplementary material for: Associations between novel anthropometric measures and the prevalence of hypertension among 45,853 adults: A cross-sectional study
Source: Front Cardiovasc Med. 2022 Nov 3;9:1050654. doi: 10.3389/fcvm.2022.1050654 (PMC9669705; doi:10.3389/fcvm.2022.1050654)
Supplement: Supplementary Table S5 — Sensitivity analysis of anthropometric measures and hypertension among participants without any medical therapy. [file Table_5.DOCX]

**Table S5. Sensitivity analysis of Anthropometric Measures and Hypertension among Participants without any Medical Therapy**

|  | Non-adjusted model |  | Model I |  | Model II |  |
| --- | --- | --- | --- | --- | --- | --- |
|  | **OR [95% CI]** | ***P* value** | **OR [95% CI]** | ***P* value** | **OR [95% CI]** | ***P* value** |
| BW | 1.61 [1.57, 1.65] | <0.001*** | 1.66 [1.61, 1.71] | <0.001*** | 1.58 [1.52, 1.62] | <0.001*** |
| BMI | 1.58 [1.54, 1.63] | <0.001*** | 1.66 [1.61, 1.71] | <0.001*** | 1.65 [1.60, 1.70] | <0.001*** |
| WC | 1.81 [1.76, 1.86] | <0.001*** | 1.65 [1.60, 1.69] | <0.001*** | 1.63 [1.59, 1.68] | <0.001*** |
| WtHR | 1.62 [1.58, 1.67] | <0.001*** | 1.66 [1.62, 1.71] | <0.001*** | 1.66 [1.61, 1.71] | <0.001*** |
| CI | 1.78 [1.73, 1.83] | <0.001*** | 1.40 [1.36, 1.44] | <0.001*** | 1.38 [1.34, 1.42] | <0.001*** |
| ABSI | 6.96 [5.28, 9.19] | <0.001*** | 1.76 [1.28, 2.42] | <0.001*** | 1.38 [1, 1.91] | <0.001*** |
| BRI | 1.68 [1.64, 1.73] | <0.001*** | 1.63 [1.59, 1.68] | <0.001*** | 1.62 [1.57, 1.67] | <0.001*** |
| LAP | 1.39 [1.36, 1.42] | <0.001*** | 1.28 [1.24, 1.31] | <0.001*** | 1.26 [1.23, 1.30] | <0.001*** |

Data are presented as OR [95% CI], and *P* values for per SD increment. Model I adjusted for age, sex, and race/ethnicity. Model II adjusted for age, sex, race/ethnicity, smoking, drinking, education levels, diabetes and eGFR. OR, odds ratio; CI, confidence interval; SD, standard deviation; BW, body weight; BMI, body mass index; WC, waist circumference; WtHR, waist-to-height ratio; CI, conicity index; ABSI, a body shape index; BRI, body round index; LAP, lipid accumulation product. *** *P* value<0.001, ** *P* value<0.01, * *P* value<0.05.
